# Aboriginal peoples’ perspectives about COVID-19 vaccines and motivations to seek vaccination: a qualitative study

**DOI:** 10.1136/bmjgh-2022-008815

**Published:** 2022-07-20

**Authors:** Simon Graham, Megan Blaxland, Reuben Bolt, Mitchell Beadman, Kristy Gardner, Kacey Martin, Michael Doyle, Karen Beetson, Dean Murphy, Stephen Bell, Christy E Newman, Joanne Bryant

**Affiliations:** 1 Department of Infectious Diseases, The Peter Doherty Institute for Infection and Immunity, Melbourne Medical School, University of Melbourne, Melbourne, Victoria, Australia; 2 Social Policy Research Centre, UNSW Sydney, Sydney, New South Wales, Australia; 3 Charles Darwin University, Casuarina, Northern Territory, Australia; 4 Centre for Social Research in Health, UNSW Sydney, Sydney, New South Wales, Australia; 5 NHMRC Centre for Research Excellence in Indigenous Health and Alcohol, Central Clinical School, Faculty of Medicine and Health, The University of Sydney, Sydney, New South Wales, Australia; 6 Department of Aboriginal Health, South Western Sydney Local Health District, Liverpool Hospital, Liverpool, New South Wales, Australia; 7 The Kirby Institute for Infection and Immunity, UNSW Sydney, Sydney, New South Wales, Australia; 8 UQ Poche Centre for Indigenous Health, The University of Queensland, Saint Lucia, Queensland, Australia; 9 School of Public Health, The University of Queensland, Brisbane, Queensland, Australia

**Keywords:** vaccines, respiratory infections, SARS, qualitative study

## Abstract

**Introduction:**

Aboriginal and Torres Strait Islander (Aboriginal) people compared with non-Aboriginal people in Australia have higher rates of chronic conditions. These conditions increase the risk of poorer health outcomes if infected with COVID-19, highlighting the importance of COVID-19 vaccination. This study examined what Aboriginal people think about COVID-19 vaccines, reasons why they were vaccinated or not vaccinated and factors involved in receiving COVID-19 vaccination.

**Methods:**

We used a participatory peer researcher method to interview 35 Aboriginal people aged 15–80 years living in Western Sydney, Australia. Local Aboriginal people who had ties with the community conducted the interviews. The questions and analyses were framed using the WHO’s Behavioural and Social Drivers of COVID-19 model. Interviews occurred between February 2021 and March 2021. Peer researchers were paid for their time in training and to conduct the interviews and each participant received $50.

**Results:**

Reasons why participants would seek vaccination included: to protect themselves from infection and severe illness, to protect others in their community, to travel again and to return to ‘normal life’. Reasons why some participants were hesitant about being vaccinated included: fear of vaccine side effects; negative stories on social media; and distrust in Australian governments and medical institutions. Aboriginal people preferred to access COVID-19 vaccination through their local Aboriginal Health Service or a general practitioner they already knew.

**Conclusion:**

Achieving high vaccination rates in Aboriginal communities is possible if vaccination programmes are delivered through trusted general practitioners or Aboriginal Health Services.

**WHAT IS ALREADY KNOWN ON THIS TOPIC**

- Achieving high rates of COVID-19 vaccination can reduce severe illness, hospitalisation and death.
- Aboriginal people have higher rates of chronic conditions, which increases their risk of poor health outcomes if infected with COVID-19.
- Reaching high rates of childhood vaccinations has been achieved among Aboriginal children.

**WHAT THIS STUDY ADDS**

- Participants trusted Aboriginal Health Services to provide information about COVID-19, COVID-19 testing and to deliver COVID-19 vaccinations.
- Misinformation online about COVID-19 and COVID-19 vaccines played a role in vaccine hesitancy.
- Motivations for vaccination included protecting themselves and others in their community/s.
- Mistrust in governments and medical institutions due to difficult histories existed.

**HOW THIS STUDY MIGHT AFFECT RESEARCH, PRACTICE OR POLICY**

- Aboriginal people valued the benefits of COVID-19 vaccines, but they raised concerns about how COVID-19 information, testing and vaccinations were delivered.
- Vaccination programmes targeting Aboriginal people should be led and delivered through Aboriginal Health Services as these are trusted sources of information and healthcare for Aboriginal people.

## Introduction

In Australia, an estimated 8.6 million cases of COVID-19 have been notified (~30% of the Australian population), with 10518 deaths (~0.1% of COVID-19 cases).^1^ Individuals over 60 years of age, those with chronic health conditions and those who are immunocompromised (such as those undergoing chemotherapy) have an increased risk of hospitalisation and death if infected with COVID-19.^2^ Aboriginal, including Torres Strait Islander (Aboriginal), people are the first peoples in Australia,^3^ and in 2021 there were ~812000 Aboriginal people (~3% of the Australian population).^4^ Higher rates of chronic health conditions such as diabetes, cardiovascular disease and hypertension are reported in Aboriginal people compared with non-Aboriginal people.^5^ This highlights that achieving high rates of COVID-19 vaccination is important to reduce severe illness and hospitalisation among Aboriginal people.^6^

The initial response to COVID-19 for Aboriginal people in Australia involved Aboriginal leaders and Aboriginal Health Services (AHS) making decisions to protect communities. These activities included Aboriginal run testing sites staffed with Aboriginal people, providing information to Aboriginal people about COVID-19, facilitating transfers to hospitals and, in remote Aboriginal communities, restricted access for visitors.^7^ These measures were informed by lessons learnt from the 2009 H1N1 influenza pandemic where the rate of diagnoses were three times greater, the rate of hospitalisations were four times greater and the rate of intensive care unit admissions were six times greater, among Aboriginal people compared with non-Aboriginal people.^8–10^ Although responses to pandemics aim to address the whole population, factors other than health influence different subpopulations’ ability to access information, testing, transportation, hospitals and treatment.^9^ These factors, including social disadvantage such as poverty, housing, education and employment, can increase inequality during and after pandemics.^11^

Australia’s colonial history has shaped the discrimination that Aboriginal people experience today.^12^ Aboriginal peoples’ history with Australian governments has been difficult with policies aiming to assimilate Aboriginal people into white society, including the stolen generations (ie, the removal of Aboriginal children from their parents and placing them with white families or institutions aimed at achieving ‘assimilation’).^13^ Discrimination and racism experienced by Aboriginal people has a broader impact than just health.^14 15^ It impacts the daily life of Aboriginal people from access to services, education and employment, which are reinforced through negative depictions of Aboriginal people in the media, including stories about high incarceration rates, deaths in custody and violence.^16 17^ Australia’s history impacts the current social and physical health of Aboriginal people and intergenerational trauma has been identified in grandparents, parents and their children.^14 18^ Aboriginal leaders in Australia have called for Aboriginal people to be in positions of power and decision making so that appropriate programmes effectively increase COVID-19 vaccination rates.^7^

In 2021, the Australian Therapeutics Goods Administration approved two COVID-19 vaccines (AZD1222 (Astra Zeneca) and Pfizer/BioTech (Pfizer)) for distribution through Australia’s universal health system.^12 19^ Two doses provide immunity against infection, and significant reductions in developing severe illness.^12 19^ Australia was late to roll out these vaccines and problems with thrombosis and thrombocytopenia from the Astra Zeneca vaccine led to Australia’s Technical Advisory Group on Immunisation recommending that Astra Zeneca no longer be distributed.^15 20^ As of January 2022, there were three COVID-19 vaccines in distribution in Australia, Pfizer, Moderna and Nuvaxovid (Novavax).^21^ In January 2022, 92% of non-Aboriginal people in Australia were fully vaccinated (defined as receiving two doses of a COVID-19 vaccine) compared with 74% of Aboriginal people.^22^ The lower uptake of COVID-19 vaccination among Aboriginal people was especially concerning at the time because herd immunity (where 80% or more are vaccinated against a disease) had not yet been achieved among Aboriginal people.^23^

Since the opening of state and territory and international borders in Australia, COVID-19 cases have increased, including in Aboriginal people, the first deaths among Aboriginal people occurred and the proportion of Aboriginal people vaccinated has continued to be significantly lower than in the non-Aboriginal population.^22^ To assist in designing targeted COVID-19 vaccination programmes to increase vaccination among Aboriginal people, our study examined Aboriginal peoples’ motivations to be vaccinated and where they would prefer to access vaccinations for COVID-19. In this paper, we describe perceptions toward vaccines from Aboriginal people living in Western Sydney, Australia. In 2020, the WHO developed the Behavioural and Social Drivers (BeSD) model of vaccination as a guide for countries to design targeted vaccination programmes for subpopulations so they could achieve herd immunity.^24^ We used the BeSD model to explore what people think and feel about vaccines, the social processes that drive or inhibit vaccination and the practical factors involved in seeking and receiving vaccination.

## Methods

### The project

The ‘Rapid qualitative assessment of COVID-19 health needs in urban Aboriginal communities in New South Wales (NSW)’ study was funded by NSW Health. It is a substudy of the larger ‘Fostering the Sexual Well-Being of Aboriginal Young People’ study, which was funded by The Australia Research Council.

### Patient and public involvement

The larger project described above commenced in 2018 and has three partner organisations: Family Planning NSW (FPNSW), Nepean Blue Mountains Local Health District and South West Sydney Local Health District. FPNSW provides social and clinical reproductive and sexual health services. The local partners had existing relationships with local Aboriginal communities, which identified and recruited local interviewers, recruited participants and conducted the interviews as part of the larger study. In the COVID-19 subproject, the local interviewers and partner organisations actively participated in the design, recruitment, hiring of local Aboriginal people, training of local Aboriginal people to conduct the interviews, the design and trialling of interview questions, and in the interpretation of the results. The substudy was based in three Aboriginal communities: two in Sydney and one in a regional town of NSW. This paper presents data from one of these sites. The other sites are not included because, for the first site, we did not collect information about vaccines as it was early in the pandemic, and for the other site, data collection was delayed due to ongoing COVID-19 lockdowns.

### Governance

The study was overseen by an investigator committee consisting of Aboriginal and non-Aboriginal academics and community representatives. An Aboriginal Research Advisory Committee was established and chaired by one of the Aboriginal Chief Investigators to oversee issues of cultural sensitivity, review and approval of ethics applications, interview questions, recruitment, data collection and dissemination of findings. Two Aboriginal people were employed to work on project management, research training, data collection and research dissemination. The study provided research training for Aboriginal people in qualitative interview methods, conducting interviews, data analyses and writing peer-review papers.

### Aboriginal data sovereignty

Our study used the principles of Aboriginal data sovereignty, which highlights Aboriginal peoples’ ‘right to maintain, control, protect and develop their cultural heritage, traditional knowledge and traditional cultural expressions, as well as their right to maintain, control, protect and develop their intellectual property over these’.^25^ We used these principles in the design of data collection through hiring local Aboriginal people, establishing an Aboriginal advisory group to oversee all aspects of the project, submitting the research design to an Aboriginal ethics committee and all analyses and publications go through an approval process that involves the Aboriginal advisory group, ethics committee clearance and the inclusion of Aboriginal people as authors (preferably as senior authors).

### Inclusion criteria

Participants needed to identify as Aboriginal and/or Torres Strait Islander, reside in the local area and be able to respond in English.

### Interviewing method

We used a peer interviewing method for data collection similar to a previous study in Aboriginal communities.^26^ The method involved training a group of eight Aboriginal young people as qualitative interviewers (five females and three males), each of whom then conducted interviews with Aboriginal people within their networks. By focusing on existing social networks, the method tapped into relationships where rapport and trust already existed. This has been shown to facilitate a more comfortable and open interviewing context and it minimised the risk of embarrassment when discussing sensitive matters.^27 28^ This also enabled Aboriginal people to drive the research on their own terms and talk about the experiences and values that were most relevant to them.^29^

Researchers conducted a 2-day training session with the eight peer interviewers. The training included information about obtaining verbal consent, voluntary participation, confidentiality and anonymity, guidelines for selecting and approaching potential interviewees, qualitative interview skills and the ethical management of data. Peer interviewers were paid at the UNSW Sydney student casual rate for their time in training and interviewing participants.

### Recruitment

The peer interviewers were recruited with the assistance of local Aboriginal Community Centres. Peer interviewers then recruited study participants. They were each asked to invite five people from their community networks (eg, friends, cousins, siblings, etc) to participate in the research. This recruitment approach has been successfully used to recruit Aboriginal people previously.^30^ AHSs are one type of local Aboriginal Community Centre that is Aboriginal-led, provides clinical and allied health services and most importantly provides a culturally safe place for Aboriginal people to access health information, clinical and allied health services. This is critical, because this increases access through: (1) responsiveness to community needs, (2) trusted relationships and (3) shared cultural background and understanding.^31^ In this study, recruiting through local community members and a local Aboriginal community centre maximised our ability to recruit Aboriginal people and create a culturally safe research environment.

The interviews occurred in safe and private locations (eg, friends houses, backyard, etc). Interviews were recorded using a digital audio recorder and transcribed by a professional transcriber working under a confidentiality agreement with the research team. Each participant received $50 for their time.

### Interview questions

Interview questions covered four topics: COVID-19 prevention (how individuals and families protected themselves, eg, by mask wearing, restricting mobility and seeking information about COVID-19), access to health services (hospitals, primary health services, AHSs and Telehealth), how families and communities were maintaining their relationships (managing the number of people allowed in your home, how people stayed connected and impact on community connections) and vaccines (views about COVID-19 vaccines, motivations to get or not get vaccinated and where to access vaccination).

This paper focuses on data collected about vaccines. The questions and analyses were framed using the WHO’s BeSD model,^24^ which is modelled from the increasing vaccination: putting psychological science into action publication.^32^ The model is designed to capture factors that increase vaccine uptake, including what people think and feel about vaccines, social processes that drive or inhibit vaccination, individual motivations and practical factors involved in seeking and receiving vaccination. The goal of the model is to support responses based on local contexts and experiences. The domains are summarised in table 1.

**Table 1:** Summary of the WHO’s Behavioural and Social Drivers of COVID-19 model

Drivers	Definition
What people think and feel	Includes data about confidence in vaccine benefits and safety, perceived risk for self and others
Social processes	Includes how and whether influential others support vaccination, vaccination norms and trust in vaccine providers
Practical issues	Includes knowing where and how to access vaccines, preferred sites for vaccination

### Data analysis

The data is stored on a secure password-protected server, accessible only by the research team. We analysed the data using thematic methods focused on the elements of the BeSD model.^24^ Interviews were transcribed then coded line by line using NVivo V.12.^33^ The coding framework included high-level codes based on the BeSD model, with subcodes developed in response to the data in order to reflect participant experiences. This approach supported deductive analysis related to vaccine topics of interest and inductive analysis regarding unanticipated findings. The coding and analysis were workshopped among the project team, checked with the project research partners, and further refined in the process of writing the paper. Such thematic analysis identified themes within and across interviews and highlighted ways in which participants understood COVID-19 restrictions, and access to health services and vaccines.^34^ The names of individuals were changed to protect their identity.

### COVID-19 context at the time of interviews

The interviews took place between February and March 2021. During this time, Western Sydney was experiencing an outbreak of COVID-19. There were heightened restrictions on the number of people who could visit households and participate in social events. While Australia had started to roll out its COVID-19 vaccination programme, only health workers, immigration staff at airports, airline staff, aged care workers, people with underlying health condition and those aged over 60 years were eligible to receive a COVID-19 vaccination. Reports of blood clots from receiving the Astra Zeneca vaccine for people under 50 years of age had not been made.

## Results

### The participants

Overall, 35 Aboriginal young people participated. Participants identified with a range of Aboriginal communities, most commonly identifying with Kamilaroi, Wiradjuri and Dunghutti. Twenty-two of the participants were women, and 21 participants were aged 16–29 years.

Three themes are presented below, related to each domain of the BeSD model.^24^ Themes identify: what people think about vaccines, indicating that they were cautious but had not arrived at a firm view; that social processes such as mistrust in government and the vaccine development were prominent; and that participants had confidence in local trusted services such as AHS, community organisations and known general practitioners.

### What people think and feel about COVID-19 vaccines

Most of the participants were cautious about vaccines but had not arrived at a firm view about whether they would be vaccinated. Key themes were related to vaccine safety and the benefits of being vaccinated.

#### Vaccine safety concerns

Participants said that they were cautious about the vaccines because they were not confident that the vaccines were safe to receive. Their comments pointed to worries that vaccines had been developed too quickly to be tested thoroughly, and that this was the reason they had heard reports of serious side effects, including death. For example:

It took 1 year to create a vaccine. I think that’s crazy. I don’t trust it. We’ve probably gotta wait for another country to see the side effects. Because I’m just scared of what may happen. (Male, 16–29 years)

[Interviewer: ‘Are you willing to get vaccinated?’] Not yet. ‘Cause I’ve heard bad stuff about it. Like apparently two people died somewhere … I’m pretty sure it was in Australia. I’m not sure but I wouldn’t get it personally because of that reason and because it’s new. (Female, 16–29 years)

These participants explain that the ‘newness’ of the vaccines meant they did not feel confident that they are safe. Having heard that people had died after being vaccinated reaffirmed their caution, and the need to better understand potential side effects.

A smaller group were concerned that the vaccines were inherently unsafe. These participants believed that vaccination involved the injection of virus particles into their body to infect them. Examples of these statements included:

I think that would kind of spook people a bit, knowing that there’s little, small bits of actual virus in there and then it could be, you know, there could be a chance you could actually get the virus. ‘Cause I heard from in the UK or something they tried a virus and the female actually got the COVID-19 from the vaccine. (Male, 16–30 years)

Personally, I’m a bit sceptical about it ‘cause you have to take a bit of the virus to make a vaccine and I’d be a bit scared about putting something I don’t know in my body. (Male, 50+ years)

With this view of vaccines, some participants were very concerned that the vaccine could make them seriously unwell, or become sick with COVID-19. Some were also worried that they would pass vaccine induced COVID-19 onto others in their communities, particularly elders.

#### Vaccine benefits

When discussing the potential benefits of vaccination, several participants said that it would both help protect themselves and others. The sense that vaccination would meet a responsibility to protect others in their communities, especially elders, was strong. For example:

Yeah, I’m willing to be vaccinated. To be honest, I think it’s important that a lot of people are vaccinated so we can get back to the normality of things. Yeah. So I’m willing to sort of speed that up and do my part. (Male, 30–49 years)

Similarly, another participant said protecting others was his main motivation for vaccination by saying, ‘Yeah, for sure. If I had to get it for my dad or my mum, or my aunties or uncles, I would get it for them. Yes’ (Male, 16–29 years).

Here, they show a strong commitment to others and a willingness to be vaccinated to protect loved ones and other community members.

As described by the participant above, a connected motivation was to help ‘get back to the normality of things’. Others also expressed a desire to be vaccinated so that life could return to ‘normal’. For younger participants, this tended to focus on travel and recreational activities with friends. An example was:

I don’t know. But then I, probably would [get vaccinated] just because I wanna get out there more, and I just wanna live my life. (Female, 16–29 years)

Another young female said:

If it means that’s vaccination is] what’s gonna get me back to travelling again and, stuff like that, well, I’ve got no choice, do I? (Female, 16–29 years)

These young women want to return to the social lives they had before the pandemic and saw the vaccine as a way to make that possible.

In contrast, older participants were more likely to describe the benefits of vaccination in relation to health, longevity and being able to stay closely connected with their families. For example:

I wanna live longer. I wanna, you know, be around my husband and my children. And I wanna see the future generation who have their own families. (Female, 50+ years)

Here, she describes the vaccine allowing her to live well into the future to stay connected with loved ones and their children.

#### Lack of trust

A key message from participants when discussing trust in vaccine processes related to a lack of trust in governments. For example, when talking about the governments’ messaging and COVID-19 vaccination roll out, several participants spoke about Aboriginal peoples’ difficult history with Australian governments, which highlighted a history of racism and mistrust. For example:

I have heard from a lot of other Aboriginal people though that are a bit wary of the vaccination. And I guess, you know, it’s something coming from the government and we’re just naturally wary of the government … For myself, like, like I’m gonna get it … but I do know there’s a, it’s a trust issue. A lot of people don’t trust the hospitals or they don’t trust the government. (Male, 30–49 years)

I do know that there is a fear about it being spread among the community members that, you know, the, especially with the Indigenous communities, remote Indigenous communities being used as test dummies. I don’t know how, how true that is but I’ve certainly heard that and I’ve heard that being a negative, you know, response to the vaccine. (Female, 50+ years)

For the above participants and others, caution about vaccine safety was tied to a profound distrust in governments and health institutions. This mistrust derives from a long history of race-based maltreatment toward Aboriginal peoples from white institutions throughout Australia’s colonial past.^17 35^

In contrast, participants expressed high levels of trust in AHSs or Aboriginal workers in other health settings.^36 37^ For example, one participant reflected the comments of many when he said that the most important consideration was trust and a known setting:

I would prefer if it was just at my general practitioner [GP]. If someone’s been seeing in a GP or going to the [Aboriginal Health Service], wherever the people are comfortable going … a lot more people will trust their local GP than they would going to some doctor in a hospital they’ve never met before … like I said, it’s a trust thing. People won’t trust taking the word of someone they’ve never met before. Whereas, if it was with their GP or their local [Aboriginal Health Service], or something like that, they would be more likely to trust it. (Male, 30–49 years)

Here, he explained that trust is particularly likely at a small clinic, like a known general practitioner or AHS, compared with an unknown practitioner in a large and unfamiliar hospital. Comments like these were expressed by many participants. While others had similar levels of trust in local community organisations who work in collaboration with health providers, for example:

But I think they’d feel more comfortable maybe [in] a place like here, like a local community centre that they know and visit often. So they’d feel more comfortable getting it done there or an [Aboriginal Health Service]. (Male, 16–30 years)

#### Practical issues

Participants’ confidence in local AHSs, community organisations and known practitioners came from past experience. They felt that these providers understood the needs of their communities and provided health services appropriately. This meant that trusted providers offer services in ways that also address practical needs. For example, a participant explained how the community centre supported influenza vaccination:

Well, we used to have like a pop-up van that used to drive around when we’d have like little events here at [the Aboriginal community service]. They would check like your, just your main health and stuff and would give the influenza vaccinations through that. I think that would be a really big like thing. We could like really help the Indigenous like communities, yeah, for sure. (Female, 30–49 years)

Another participant described similar approaches to facilitating hepatitis B vaccination by community centres. These and other participants noted a need for transport, so offering practical support in the form of a bus facilitated access to vaccines. As one participant explained, these services felt comfortable, familiar and safe:

[Aboriginal Health Service] It’s just like your people. Like it’s a place for your people to go to. You’re more comfortable. (Female, 16–29 years)

## Discussion

Aboriginal people living in Western Sydney had various reasons to be vaccinated ranging from protecting themselves and others from severe illness, to being able to travel and return to ‘normal life’. Reasons for being hesitant about COVID-19 vaccination ranged from fear of side effects to distrust in the Australian government and medical institutions. Trust in those providing the vaccine played a key role in the decision-making of the participants for where they preferred to be vaccinated, with local AHS and going to a known general practitioner (GPs) being the preferred access points.

Some participants in our study expressed concern in the side effects from COVID-19 vaccines and said that they would wait and see what happens before deciding whether to be vaccinated. These views have also been expressed by non-Aboriginal people in Australia and overseas.^38^ A review of COVID-19 hesitancy from high-income countries, highlighted that those who are hesitant were more likely to have a history of not receiving the influenza vaccine, to have a lower self-perceived risk of contracting COVID-19, to not fear COVID-19, to believe that COVID-19 is not a severe disease and to not have chronic medical conditions.^38^ To successfully increase the vaccination rate among Aboriginal people in Australia, vaccination programmes should be delivered through trusted service providers such as an AHS or local GPs. If a town does not have an AHS, then support for local GPs who provide health services to local Aboriginal communities should be provided to facilitate access to the COVID-19 vaccine. If GPs are providing health services to local Aboriginal communities, they would benefit from hiring local Aboriginal people to help design, distribute and connect with local communities to provide vaccine information and deliver vaccinations.

Hesitancy about COVID-19 vaccines has been heightened by misinformation. Two examples of misinformation about COVID-19 to Aboriginal communities have been documented.^38 39^ The first was in Western Australia, where a prayer group distributed information stating that God would protect them against COVID-19. The second was in the NSW Aboriginal community of Wilcannia during an outbreak, where misinformation was spread regarding treatments of COVID-19, and which discouraged vaccination.^39^ Accurate and evidence-based information about COVID-19 vaccines is needed so that Aboriginal people can make informed decisions about being vaccinated for COVID-19. These evidence-based health messages are also best delivered by Aboriginal people to Aboriginal people.^40^

Some participants in our study discussed distrust in Australian governments and medical institutions as one of the reasons they were hesitant about vaccines and in particular the COVID-19 vaccines. They contextualised this in a long history of discrimination and racism experienced by Aboriginal people when accessing mainstream health services such as hospitals.^17^ Racial minorities living in wealthy countries outside of Australia who have lower COVID-19 vaccination rates compared with their white counterparts have also spoken about distrust in governments and medical institutions due to long histories of racism.^41^ Concerningly, if Aboriginal people are more likely to have underlying chronic health conditions, are not vaccinated and are infected with COVID-19, they will also be more likely to need to access a hospital. In this situation, they would face a dilemma: either attend a hospital where they have had negative experiences or avoid hospitals and risk severe illness or possible death. There is a dire need to address discrimination and racism experienced by some Aboriginal people when accessing hospitals, especially in an intense environment such as the emergency department.

Distrust in Australian governments and medical institutions can be influenced by a lack of inclusion of local Aboriginal communities in health provision and planning or from a lack of knowledge among health providers regarding Aboriginal communities and how to deliver their health services. One example, which occurred after the first wave of data collection, was in the Western NSW Aboriginal community of Brewarrina. To increase COVID-19 vaccination rates in the local Aboriginal community, the state government set up a vaccination hub; however, the hub only supplied the Astra Zeneca vaccine and, due to the reported blood clotting issues, the local community were hesitant to be vaccinated.^39^ If local Aboriginal people and leaders were involved in the design of the vaccination hub, then this mistake could have been avoided. This highlights that context and inclusion of Aboriginal people in the design and delivery of vaccination programmes is important to build trust if high vaccination rates are to be achieved.

### Limitations and strengths

Aboriginal people in this study were recruited in Western Sydney, and as a result our sample may not be generalisable to other areas of Australia, or to other Indigenous communities in other countries. We acknowledge that the timing of the interviews may have influenced the responses from participants, and some participants’ views might change, for example, in response to further outbreaks or if they knew someone who needed to go to a hospital because of COVID-19. As most of our participants were female, if we were to interview a larger cohort, we could have strengthened our analysis and gained more insight by including the views of men, people living with chronic conditions and Elders.

### Recommendations and future research

Our data indicated that Aboriginal people value the benefits of COVID-19 vaccines but were seeking more information. It is promising in Australia that, for other infections where a vaccine is available, high vaccination rates of 91% have been achieved, including among Aboriginal children.^42^ This demonstrates that high vaccination rates are possible if Aboriginal people and local communities are included in the design, approach and implementation of vaccination programmes. A key part of this is the involvement of AHSs, as they are a preferred way Aboriginal people want to access information, ask questions and then make decisions about whether to be vaccinated.^40^

Trust and feeling safe with the AHS played a strong role. Although there are AHSs in each state and territory in Australia, not all Aboriginal communities have a local AHS. This suggests that mainstream health services that aim to provide services to Aboriginal people need to increase trust and provide a safe environment for Aboriginal people. This could be achieved through hiring Aboriginal people as Aboriginal liaison officers to assist Aboriginal patients, and second, implement Aboriginal cultural safety training for non-Aboriginal staff. These are positive responses to help address racism and discrimination in the healthcare system.

In designing programmes that reduce racism and discrimination in Australia, a key part of future policy development, programmes and research is Aboriginal leadership. When Aboriginal people and communities are in control of the design, messaging and delivery of community-based programmes, such as COVID-19 testing or vaccination, then access increases and most importantly Aboriginal people feel safe to access these services.^7^ Seeing other Aboriginal people at mainstream services as staff, and especially as senior staff in positions of power, could also be a way to increase access to vaccination and, more broadly, make hospitals a more culturally safe place.^43^ Although there is much diversity within Aboriginal populations in Australia and Indigenous populations globally, we do have a shared history of colonisation and discrimination.^44 45^ Our research may be of use to other Indigenous populations, both in the study design and our finding that Indigenous communities value Indigenous designed and delivered COVID-19 vaccination programmes.

## Conclusion

We found that Aboriginal community views toward COVID-19 vaccination were mixed. Trust in those administering the vaccine and feeling safe when accessing COVID-19 testing and vaccination was important to Aboriginal people. There was a preference for accessing testing and vaccination at an AHS or a trusted local GP. Developing and implementing programmes that reduce discrimination and racism experienced by Aboriginal people when accessing mainstream health services are clearly needed.

## Data Availability

Data are available upon reasonable request.
